# Psycholinguistic and socioemotional characteristics of young offenders: Do language abilities and gender matter?

**DOI:** 10.1111/lcrp.12150

**Published:** 2019-04-05

**Authors:** Maxine Winstanley, Roger T. Webb, Gina Conti‐Ramsden

**Affiliations:** ^1^ Division of Human Communication, Development and Hearing School of Health Sciences The University of Manchester UK; ^2^ Manchester Academic Health Sciences Centre (MAHSC) UK; ^3^ Centre for Mental Health and Safety Division of Psychology and Mental Health School of Health Sciences The University of Manchester UK

**Keywords:** offending, young adults, developmental language disorders, psycholinguistic profiles, socioemotional characteristics, police contact

## Abstract

**Purpose:**

Previous research demonstrates an association between developmental language disorder (DLD) and criminal offending. International research also implicates alexithymia as being over‐represented in forensic samples. This study provides a comprehensive examination of the psycholinguistic and socioemotional profiles of males and females in the youth justice system, with a focus on first‐time entrants. In the context of restorative justice (RJ) underpinning youth justice disposals, this allows for informed intervention and identifies those who may be compromised in their ability to effectively engage in certain interventions.

**Methods:**

Participants (*N *= 145) from a triage centre and youth offending teams, with a mean age of 15.8, completed a range of standardized psycholinguistic assessments considering non‐verbal IQ, expressive and receptive language measures, and literacy. Additionally, socioemotional measures completed included The Alexithymia Scale and the Strengths and Difficulties Questionnaire.

**Results:**

Developmental language disorder was present in 87 participants. Except for the emotional score, no statistically significant gender differences were found. The mean language scores for the DLD group were more than 2.25 standard deviations below the normative mean, and they demonstrated greater literacy difficulties. A high proportion of the group met the criteria for alexithymia/possible alexithymia (60%), and this was not associated with DLD.

**Conclusions:**

High prevalence values for DLD and socioemotional difficulties, not generally gender‐specific, were found. These difficulties have the possibility to compromise a young person's ability to engage in rehabilitative strategies. Language assessment and identification of difficulties, especially DLD, upon entry to the youth justice service, would assist when planning interventions.

## Background

Youth offending is a serious problem that is costly to society (Romeo, Knapp, & Scott, 2006), degrades local environments, and can evoke fear in citizens (Jacobson & Kirby, 2012). Careful consideration and an understanding of the correlates of youth offending, including the psycholinguistic and socioemotional characteristics of the young people involved in offending, are, therefore, warranted. In particular, the role of language not only contributes to this characterization but also provides an approach for identifying clusters of difficulties in the profiles of young offenders with and without developmental language disorder (DLD). Such knowledge can inform policy as well as practice, in particular those involved in the planning and development of rehabilitation strategies.

Developmental language disorder refers to significant, persistent problems understanding and/or using spoken language that are not attributable to other difficulties such as hearing impairment or autistic spectrum disorder (Bishop, Snowling, Thompson, & Greenhalgh, 2016; Bishop, Snowling, Thompson, Greenhalgh, & Consortium, 2016). Recent evidence has highlighted an association between offending and DLD, which persists even after controlling for potential confounders such as socio‐economic position and years of schooling (Bryan, Garvani, Gregory, & Kilner, 2015; Hopkins, Clegg, & Stackhouse, 2018; Snow & Powell, 2008). The deficits youth offender samples display in language‐based tasks have covered all domains of language, including receptive, expressive, and figurative (Bryan *et al*., 2015; Snow, Woodward, Mathis, & Powell, 2016). Additionally, these language‐based tasks have considered the form, content, and use of language from word (Lount, Purdy, & Hand, 2017) to sentence level (Bryan, Freer, & Furlong, 2007) and then to extended discourse including narrative (Snow & Powell, 2005) and expository discourse (Hopkins *et al*., 2018). It has consequently been demonstrated that approximately 50% of young offenders have language deficits that would warrant a diagnosis of DLD, but which have previously gone unrecognized (Gregory & Bryan, 2011; Snow & Powell, 2011). Most of the studies originating in the United Kingdom have concentrated on incarcerated samples or those on intensive orders (Bryan *et al*., 2007; Gregory & Bryan, 2011) which are usually reserved for prolific offenders. There is a paucity of research considering young offenders early in their offending trajectory. The exception to this is a study from Hopkins *et al*. (2018) who investigated an opportunity sample of 52 young offenders. Although the young people were serving court orders for their offences, ranging in length from 4 to 18 months, it was unknown whether this was their first contact with the youth justice service (YJS). Youths with numerous offences may be more likely to exhibit DLD due to a reduced efficacy in rehabilitative methods utilized earlier on in their offending journey (Snow & Powell, 2008). In this study, we determine the language abilities of a group of young first‐time offenders on community orders in the north‐west of England. In order to increase comparability with previous research and provide a fuller picture of the young person, non‐verbal abilities were also examined. Community orders refer to interventions, aimed at reducing reoffending, delivered in the community.

Research has supported the link between early oral language skills and later reading ability (Oakhill & Cain, 2012), and children with language difficulties at school entry are at risk of literacy difficulties (Catts, Fey, Tomblin, & Zhang, 2002). Literacy skills are required to access academic aspects of school, and academic difficulties can be a risk factor for disengaging with education and engaging with disaffected peers (Gifford‐Smith, Dodge, Dishion, & McCord, 2005). Difficulties with reading have been linked with behavioural problems in childhood in both the conduct and hyperactivity domains (Maughan, Pickles, Hagell, Rutter, & Yule, 1996; St Clair, Pickles, Durkin, & Conti‐Ramsden, 2011; Tomblin, Zhang, Buckwalter, & Catts, 2000). Snowling, Adams, Bowyer‐Crane, and Tobin (2000) considered the literacy skills of 91 incarcerated young offenders in the United Kingdom with a mean age of 16 and found the young offenders performed at a mean reading age of 11.3 significantly below their chronological age and that of a control group recruited from local schools but not matched on socio‐economic status. Additionally, reading comprehension has been noted as a predictor of recidivism in a group of incarcerated youths aged 16–19 (Rucklidge, McLean, & Bateup, 2013). A low level of literacy may limit a young person's ability to access formal youth justice documentation including behaviour contracts, referral order agreements, and appointment letters. In this study, we aimed to describe in detail the reading skills of a group of young first‐time offenders.

Childhood conduct problems have also been associated with adult criminality (Moffitt, Caspi, Harrington, & Milne, 2002), and behavioural difficulties in childhood and adolescence often precede contact with the YJS. The literature pertaining to the prevalence of DLD in children exhibiting conduct problems is similar to the youth offending literature with concerns being raised regarding the referral of children to services that target the visible externalizing behaviour problems with little consideration given to underlying language abilities (Cohen *et al*., 1998). It is now well established that children with DLD are at increased risk of experiencing social, emotional, and behavioural difficulties (Durkin & Conti‐Ramsden, 2010). In a longitudinal study by Beitchman *et al*. (1996), lower scores on expressive and receptive language measures identified the children with the highest probability of developing internalizing and externalizing disorders. A recent meta‐analysis reported that children with a history of DLD were almost twice as likely to meet the criteria for internalizing problems (anxiety and depression) and over twice as likely to meet criteria for an externalizing problems (conduct disorder and attentional problems) than their typically developing peers (Yew & O'Kearney, 2013). In contrast, the presence of prosocial traits, as measured by the Strengths and Difficulties Questionnaire (SDQ; Goodman, 1997), is considered a protective factor for young people with DLD (Mok, Pickles, Durkin, & Conti‐Ramsden, 2014). The SDQ prosocial subscale consists of five positive items: ‘considerate of other people's feelings’, ‘shares readily with other children’, ‘helpful if someone is hurt, upset or feeling ill’, ‘kind to younger children’, and ‘volunteers to help others.’ Prosocial behaviour has been identified to have a strong negative correlation with behavioural difficulties in children with DLD (Farmer, 2000). In this study, we examine both externalizing and internalizing difficulties of a group of young first‐time offenders, in addition to prosocial behaviours.

As part of the Crime and Disorder Act of 1988 youth offending teams (YOTs) were established in every local authority in England and Wales between 1998 and 2000 (Youth Justice Board [YJB], 2010). Staff within YOTs oversee young people on a wide range of pre‐court and post‐court disposals as well as providing a triage service aimed at prevention. Unless they have committed a very serious offence, young first‐time offenders are typically subject to diversionary approaches, known as triage, that aim to make up for harm caused and address their offending behaviour. This allows young people who have committed a minor first offence to be diverted from the formal YJS. Irrespective of the order, the YJB have detailed that restorative justice (RJ) should be considered as an underlying principle for all youth justice disposals (YJB, 2011). An element of its appeal is that it is a theoretically grounded concept (Angel *et al*., 2014), with roots in the interaction theory of Collins (2004), Braithwaite's Reintegrative Shaming theory (1989), and Tyler's theory of procedural justice (1990). Each theory orientates to a particular aspect of RJ, with Collins (2004) suggesting that successful rituals consist of a shared focus of attention and mutual understanding therefore creating feelings of solidarity. Braithwaite (1989) offered an alternative to punitive measures of crime control by advocating prosocial strategies focussing on repair, and Tyler (2000) suggested that compliance with orders is increased if offenders deem the process of any sanction as fair, trustworthy, and non‐biased. Often seen as a practice that links an offender to a pathway towards redemption (Sherman & Strang, 2012), RJ is described as ‘a process whereby all parties with a stake in a particular offence come together to resolve collectively how to deal with the aftermath of the offence and its implications for the future’ (Marshall, 1996:37). In contrast to conventional courts, RJ is a verbally mediated process and can be described as ‘emotionally intense’ (Angel *et al*., 2014); therefore, a certain level of socioemotional and linguistic competence is required.

Young people who demonstrate language skills across multiple domains that fall well below what would be expected from their age and IQ may be compromised in their ability to effectively engage in RJ processes. Furthermore, socioemotional abilities, such as alexithymia, may also be implicated in one's ability to participate in rehabilitations with RJ principles at the centre. Alexithymia refers to a diminished ability to recognize and interpret emotions (Manninen *et al*., 2011) and an externally orientated cognitive style (Nemiah, Freyberger, & Sifneos, 1976). When considering a forensic population, Zimmermann (2006) compared juvenile offenders with demographically matched non‐offenders when investigating the associations of alexithymia and delinquency in male adolescents. Regression analysis revealed that alexithymia alone, as measured by a self‐report questionnaire, was a significant predictor of offender group membership (Zimmermann, 2006). Despite further personality and anxiety measures being added to the model, these did not reach significance. The author reported that the best goodness‐of‐fit statistic, with an overall correct classification result of 72%, included only alexithymia and family functioning. Given that the main elements of alexithymia include a difficulty in labelling emotions and expressing them to others, it is logical to suggest that language difficulties may be implicated in the causal pathway. Conversely, alexithymic individuals display emotion processing difficulties on both verbal and non‐verbal tasks (Wagner & Lee, 2008). Studies including individuals with acquired language difficulties (Henry, Phillips, Crawford, Theodorou, & Summers, 2006) have found performance on language measures, such as verbal fluency, associated with difficulties identifying feelings. Similarly, an association between delayed early speech and elevated risk of alexithymia later in life has been reported among individuals with DLD (Karukivi *et al*., 2012). A recent study found alexithymia present, or likely to be present, in 59% of a sample consisting of 100 incarcerated young offenders (Snow *et al*., 2016). Despite alexithymia being associated with poor mental health, it was not correlated with language difficulties in this sample. In this study, we investigate alexithymia in a group of young first‐time offenders.

The growing literature concerning DLD and offending populations has predominately reported on male samples (Bryan *et al*., 2007; Snow & Powell, 2008). It is well established that a higher proportion of males come into contact with youth justice (Ministry of Justice, 2018) compared to females. Therefore, in cases whereby females have been included, the proportion of females has been small. This has led to researchers undertaking analysis on the sample as a whole, regardless of gender (Gregory & Bryan, 2011; Hopkins *et al*., 2018). There are some exceptions which provide interesting preliminary evidence that there may be gender differences in the language profiles of females versus male youth offenders. For example, a cross‐sectional study examining language, emotion recognition, and mental health of 15 young female offenders on community orders in Australia found that four participants met the study's definition of DLD (Snow *et al*., 2016). Although this study consisted of a small sample size, it supported earlier findings from Sanger, Creswell, Dworak, and Schultz (2000) in the United States, who considered 78 incarcerated female offenders with a mean age of 16. The authors found that 22% of the sample scored at least 1.3 standard deviations below the mean on the Clinical Evaluation of Language Fundamentals 3 (CELF‐3; Semel & Secord, 2000). Interestingly, when the same authors considered a more comprehensive battery, their results revealed that participants were unable to provide synonyms for words such as ‘penalty’ and ‘justify’ and they could not adequately define terms such as ‘competent’, ‘caution’, or ‘priority’ (Sanger, Moore‐Brown, Montgomery, Rezac, & Keller, 2003). However, this preliminary evidence has not been replicated in other studies that have specifically looked at language differences. A study considering the language differences between adjudicated and non‐adjudicated adolescents, for example, reported a between‐group difference but no within‐group gender differences (Blanton & Dagenais, 2007). In order to provide further insight into potential gender differences in youth offenders and address the available mixed evidence based on very small sample sizes in previous studies, we include, to the best of our knowledge, the largest sample of female young offenders in the United Kingdom (UK), to provide evidence of the point prevalence of DLD in females and examine gender differences in the psycholinguistic and socioemotional profiles of young offenders.

Profiling psycholinguistic and socioemotional abilities of young people in the YJS and determining key difficulties can be useful for both prevention and informing interventions that target specific needs. Most of the research in this area has focused on samples on custodial sentences (Bryan *et al*., 2015; Snow *et al*., 2016). In this investigation, we focussed on first‐time offenders aiming to provide novel data on young people who have their first contacts with the justice system.

Specifically, this study addresses three research questions in relation to a group of 145 young offenders attending community youth offending services in the North West of England:

1. What is the context of young offenders? Offence characteristics, socio‐economic status, and educational attainment in young offenders new to the YJS.
2. Are there gender differences in (a) the prevalence of DLD and (b) psycholinguistic and socioemotional characteristics of young offenders?
3. Is there a distinct profile of psycholinguistic and socioemotional difficulties of young offenders with and without DLD?

## Method

### Ethics

This study received ethical approval from The University of Manchester, UK, and informed written consent was gained from all participants. Due to the vulnerability of the young people, consent was also obtained from a parent or a guardian, and with the approval of the ethics committee, the young offenders’ case worker was permitted to act *in loci parentis*. The caseworker was therefore provided with a copy of the information sheet and asked to read it with the young person.

### Participants and procedure

The sample included 145 young people, 96 of whom were first‐time entrants into the YJS in the North West of England. In an attempt to make the research representative of young people in the YJS, no inclusion criteria were stipulated other than having English as one's first language. The mean age of the participants was 15.8 (*SD *= 1.5), and their ages ranged from 12 to 17 years. The majority of the sample (*n* = 112) was male (33 female). This is reflective of the latest national results published in 2017, which detail that 80% of first‐time entrants into the YJS are male and their mean age is 15.2 (YJB, 2018).

Participants were tested over 1 or 2 sixty‐minute sessions at which parents and youth offending team staff were encouraged to attend. Further information on recruitment and the procedures is provided in the Supporting Information document.

### Measures

Psycholinguistic, socioemotional, and context measures were obtained (further details of each of the measures and statistical analysis used are provided in the Supporting Information document).

### Standardized psycholinguistic measures

Non‐verbal IQ (NVIQ) was assessed using the performance subscale of The Wechsler Abbreviated Scale of Intelligence (WASI, Wechsler, 1999). For language abilities, ‘formulated sentences’ (FS) and the ‘understanding spoken paragraphs’ (USP) subtests were administered. These combined subtests yielded a core language score. For ascertaining DLD status, and to avoid overdiagnosis, we followed the recommendations made by Spencer, Clegg, and Stackhouse (2012), who specified a score of 1.5 *SD* below the normative mean on the CELF‐4 subscales to determine the frequency of unidentified DLD. Reading was measured using two tests: The Test of Word Reading Efficiency – Second Edition (TOWRE–2; Torgeson, Wagner, & Rashotte, 1999) to assess ability to read printed words and Wechsler Individual Achievement Test (WIAT‐II; Wechsler, 2005) to evaluate reading comprehension.

### Socioemotional measures

The Alexithymia Scale (TAS‐20; Bagby, Taylor, & Parker, 1994) was used. The TAS‐20 is a 20‐item self‐report scale measuring (1) difficulty describing feelings (‘It is difficult for me to find the right words for my feelings’); (2) difficulty identifying feelings (‘When I am upset I don't know if I'm sad, frightened or angry’); and (3) externally orientated thinking (‘I prefer to just let things happen, rather than understand why they turned out that way’). Items are rated using a 5‐point Likert scale whereby 1 = ‘strongly disagree’, 2 = ‘disagree’, 3 = ‘neutral’, 4 = ‘agree’, and 5 = ‘strongly agree’. Scores equal to or less than 51 = non‐alexithymia, 52–60 = possible alexithymia, and scores equal to or greater than 61 = alexithymia. The Strengths & Difficulties questionnaire (SDQ; Goodman, 1997) was used to examine internalizing and externalizing difficulties, specifically, conduct problems (e.g., ‘I get very angry’), hyperactivity (e.g., ‘I am easily distracted’), emotional difficulties (e.g., ‘I worry a lot’), peer relation problems (e.g., ‘I am usually on my own’), and prosocial behaviour (e.g., ‘I try to be nice to others’). The latter scale, the prosocial scale, measures positive functioning (as opposed to difficulties). For each item, the young person could tick either ‘not true’, ‘somewhat true’, or ‘certainly true’, which reflect a score of 0, 1, and 2, respectively.

### Contextual measures

The Index of Multiple Deprivation (IMD) was applied as an ecological measure of socio‐economic position. This is a residential postcode‐based measure of area‐level deprivation that represents the immediate locality of a person's household and is calculated as a composite of the following seven domains of deprivation: income; employment; health and disability; educations skills and training; barriers to housing and services; crime; and living environment (McLennan *et al*., 2011). The higher the score, the greater the deprivation and overall the IMD can be divided into quintiles, with quintile 1 being the least deprived localities and quintile 5 the most deprived.

Detailed scrutiny of departmental files in each YOT and the triage centre took place. This was carried out to ascertain the nature of the offence the young person had committed as well as educational attainment (literacy and numeracy).

### Statistical analysis

All statistical analyses were conducted in SPSS 22 (IBM Corp. Released 2013. IBM SPSS Statistics for Windows, Version 22.0. Armonk, NY: IBM Corp), and a two‐tailed significance level of *p *=* *.05 was used unless otherwise specified. Independent *t*‐tests for continuous variables, and chi‐squared (χ^2^) tests for categorical variables, were used to compare group differences.

## Results

We provide descriptive details regarding the group before addressing each of the research questions.

**Figure 1:**
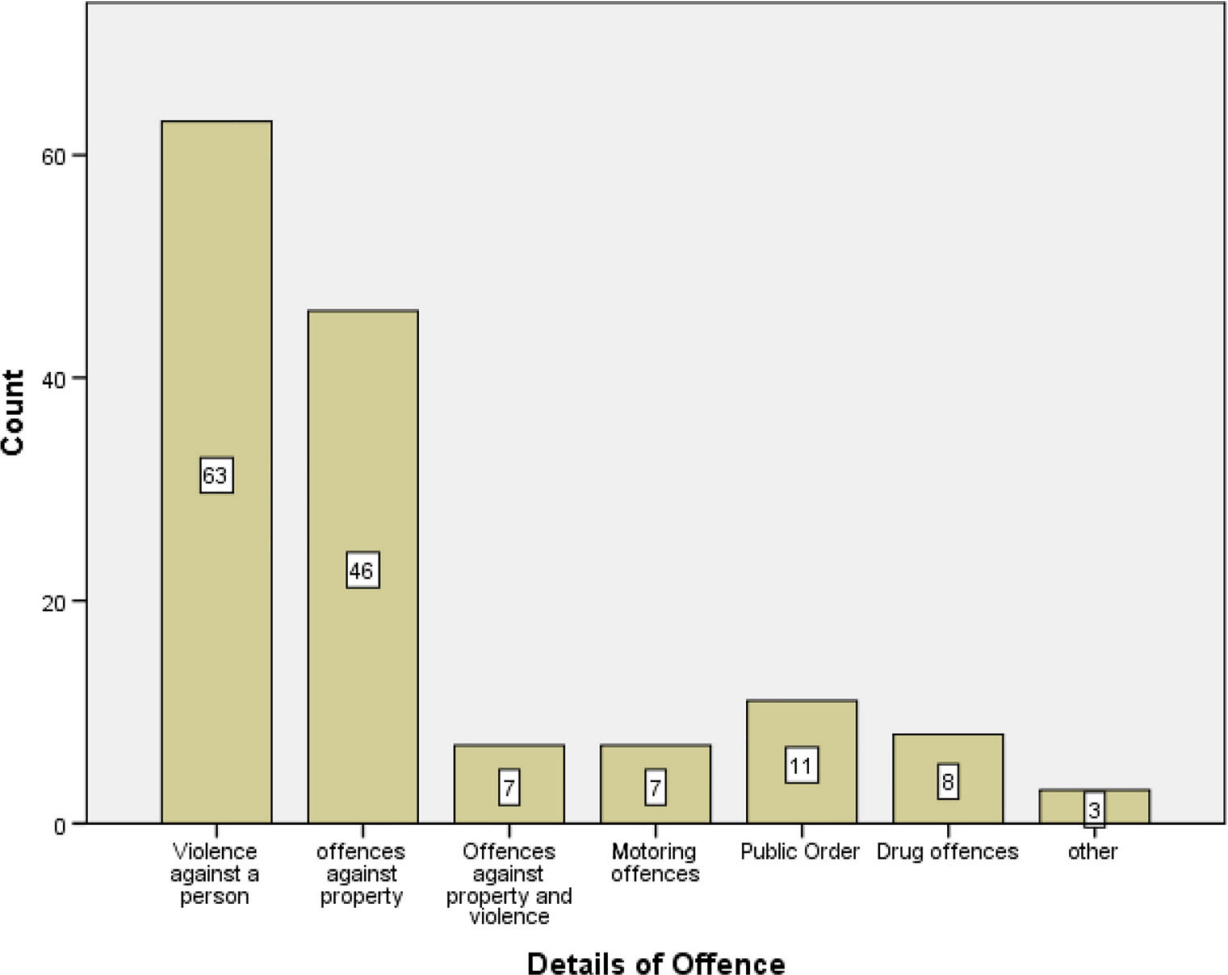
Offence type pertaining to young person's current order. [Colour figure can be viewed at wileyonlinelibrary.com]

All young offenders were either subject to triage intervention (49) or on community orders (96), with a duration ranging from 1 week to 24 months (median = 6, interquartile range = 9). Mutually exclusive offence categories committed by participants are displayed in Figure 1. In terms of socio‐economic position, the majority, 66% (95), of participants resided in quintile 5; the most deprived areas, 22% (32), were from quintile 4; and the remaining participants, 12% (18), were distributed in quintiles 1–3. Table 1 presents participants’ levels of attainment for numeracy and literacy. These data were not available for young people in the triage system, nor were they available for all the young people accessed via the YOTs. Where data from records were available, over half the young offenders had poor attainment in literacy (53%) and numeracy (54%).

**Table 1:** Attainment in literacy and numeracy for the participants on entry to the youth justice service

	Literacy (*n*)	Per cent	Numeracy (*n*)	Per cent
Below entry level	12	17	14	19
Entry level 1 Expected by age 7	13	18	10	14
Entry level 2 Expected by age 9	8	11	8	11
Entry level 3 Expected by age 11	5	7	7	10
Level 1 GCSE D‐G	23	32	24	33
Level 2 GCSE A*‐C	11	15	9	13
Total	72	100	72	100

Note

Based on available data (72 of the participants), approximately half the sample had not reached entry level 3 for numeracy and literacy expected by age 11 years.

### Are there gender differences in the characteristics and DLD status of young offenders?

As evidenced in Table 2, there were virtually no statistically significant gender differences in the profiles of males versus females in the sample (with the exception of the emotional score indicating more difficulties among females). In terms of prevalence of DLD, no significant gender difference was evident: χ^2^ (1) = 7.91, *p *=* *.37. Of the 112 males in the study, 65 (58%) met the criteria as did 22 (67%) of the females. The young people showed a variety of different language profiles with the majority, 55 participants (38%), gaining scores indicative of having both expressive and receptive DLD. A smaller proportion, 12 participants (8%), displayed receptive difficulties only, and 20 participants (14%) gained scores indicative of difficulties with the expressive domain. Given the gender similarities and the presence of DLD in nearly two thirds of the sample, further analyses were carried out for the whole sample comparing those with and without DLD.

**Table 2:** Psycholinguistic and socioemotional profiles of male versus female young offenders

	Gender	*n*	*M* (*SD*)	*t*	*p*	Mean difference	95% CI
Non‐verbal IQ	Male	110	87.6 (13.0)	0.97	.33	2.30	−2.60, 7.60
	Female	31	85.2 (11.1)				
Expressive language	Male	112	77.2 (14.2)	0.61	.55	1.73	−3.91, 7.38
	Female	33	75.4 (15.2)				
Receptive language	Male	112	77.4 (15.4)	0.17	.87	0.52	−5.53, 6.55
	Female	33	76.9 (15.5)				
Sight word reading	Male	76	83.0 (8.6)	−0.20	.84	−0.42	−4.53, 3.70
	Female	24	83.4 (9.5)				
Phonemic decoding	Male	75	88.1 (12.1)	−0.42	.68	−1.16	−6.66, 4.33
	Female	24	89.3 (10.5)				
Total word reading	Male	75	82.9 (11.5)	−0.24	.81	−0.65	−5.97, 4.57
	Female	24	83.6 (11.3)				
Reading comprehension	Male	44	81.7 (14.9)	−0.19	.85	−0.82	−9.44, 7.80
	Female	14	82.6 (10.7)				
Alexithymia (total Tas‐20)	Male	86	54.8 (11.5)	−1.54	.12	−4.18	−9.41, 1.06
	Female	25	58.9 (12.0)				
Difficulty identifying feelings	Male	86	16.3 (6.3)	−1.50	.14	−2.17	−5.03, 0.68
	Female	25	18.4 (6.4)				
Difficulty describing feelings	Male	86	14.0 (4.4)	−1.75	.08	−1.83	−3.91, 0.25
	Female	25	15.9 (5.5)				
Externally orientated thinking	Male	85	24.8 (4.7)	0.18	.87	0.11	−1.95, 2.17
	Female	25	24.6 (4.12)				
Total SDQ	Male	97	15.1 (5.8)	−1.62	.11	−1.95	−4.34, 0.43
	Female	29	17.0 (5.3)				
Emotional difficulties	Male	97	3.0 (2.4)	−2.75	.01**	−1.45	−2.50, −0.40
	Female	29	4.5 (2.9)				
Conduct problem	Male	97	3.8 (2.3)	−0.11	.91	−0.48	−0.89, 0.80
	Female	29	3.9 (1.6)				
Hyperactivity	Male	97	6.0 (2.4)	0.05	.96	0.02	−0.96, 1.00
	Female	29	6.0 (2.2)				
Peer problems	Male	97	2.3 (1.5)	−1.41	.16	−0.44	−1.05, 0.18
	Female	29	2.8 (1.5)				
Prosocial behaviours (positive scale)	Male	97	6.9 (1.8)	0.29	.77	0.11	−0.64, 0.86
	Female	29	6.8 (1.7)				

Note

**p = <.01.

### Characteristics of young offenders with and without DLD

Statistically significant between‐group differences were found on all the psycholinguistic measures (see Table 3). All scores for the non‐DLD group were in the low average, a standard score between 85 and 89 (sight word reading), to average range, a standard score between 90 and 110. The mean language score for the young offenders with DLD was more than 2.25 standard deviations below the mean, which is a standard score of <0.68. As a group, young offenders with DLD had significantly greater problems with all aspects of reading than those without DLD. In contrast, no significant differences were found between young offenders with and without DLD for alexithymia. This was the case when comparing continuous scores as well as when comparing alexithymia status categorically as presented in Table 4. The mean score for alexithymia for the group as a whole was 55.7 (*SD* = 11.7) and 60% of the group met the criteria for alexithymia/possible alexithymia. Similarly, no significant differences were found between young offenders with and without DLD for internalizing and externalizing difficulties and prosocial behaviours as measured by the SDQ (Table 5). The mean total SDQ score for the group as a whole was 15.53 (*SD* = 5.73), and 54% of the sample had total difficulties scores in the borderline/abnormal range.

**Table 3:** Psycholinguistic profiles of DLD versus non‐DLD young offenders

	Group		*t*	*df*	Mean difference	[95% CI]	Cohen's *d*
	DLD (*N *=* *87)	Non‐DLD (*N *=* *58)					
Non‐verbal IQ	83.1 (12.1)	93.2 (11.1)	5.01***	139	10.1	[6.1, 14.0]	0.8
Expressive language FS	67.8 (10.1)	90.3 (8.0)	14.3***	143	22.7	[19.6, 25.7]	2.5
Receptive language USP	67.8 (10.9)	91.4 (8.8)	13.8***	143	23.6	[20.2, 27.0]	2.4
Sight word reading efficiency	81.6 (7.92)	85.4 (9.68)	2.15*	98	3.8	[0.3, 7.3]	0.4
Phonemic decoding	86.3 (11.6)	91.7 (11.2)	2.27*	97	5.4	[0.7, 10.1]	0.7
Total word reading	80.9 (10.8)	86.6 (11.5)	2.50**	97	5.7	[1.2, 10.3]	0.5
Reading comprehension	75.7 (12.5)	88.2 (12.4)	3.81***	56	12.5	[5.9, 19.0]	1.0

Notes

All scores are standard score means and in brackets standard deviations (*SD*). All standard scores have a normative mean of 100 and a *SD* of 15. For example, a mean score for non‐verbal IQ of 83.1 for the DLD group is 1.25 *SD* below the normative mean; in the same vein, a non‐verbal IQ of 93.2 in the non‐DLD group is 0.5 *SD* below the normative mean.

DLD = developmental language disorder; FS = formulated sentences; USP = understanding spoken paragraphs.

*p < .05; **p < .01; ***p < .001.

**Table 4:** Alexithymia Scale (TAS‐20) findings by developmental language disorder (DLD) status

	Group		*t*	*p*
	DLD	Non‐DLD		
	Mean (*SD*)	Mean (*SD*)		
	*N *=* *58	*N *=* *53		
TAS 20 total score	56.6 (12.6)	54.6 (10.7)	−0.91	.36
Difficulty identifying feelings score	17.5 (7.0)	16.0 (5.6)	−1.20	.23
Difficulty describing feelings score	14.3 (5.0)	14.7 (4.3)	0.43	.67
Externally orientated style of thinking	25.3 (4.9)	24.0 (4.0)	−1.57	.12
Alexithymia status^a^				
Alexithymia (scores > 61)	24 (41%)	17 (32%)		
Possible alexithymia (52–60)	12 (21%)	13 (25%)		
Non‐alexithymia (<51)	22 (38%)	23 (43%)		

Note

a No significant group difference was found, χ^2^ (2[111] = 1.03, *p *=* *.60).

**Table 5:** Strengths & Difficulties Questionnaire (SDQ) results by developmental language disorder (DLD) status

	Group		*t*	*p*
	DLD	Non‐DLD		
	Mean (*SD*)	Mean (*SD*)		
	*N *=* *71	*N *=* *55		
SDQ total score	16.1 (5.6)	14.8 (5.8)	−1.30	.19
Emotional difficulties	3.5 (2.7)	3.1 (2.4)	−0.86	.39
Conduct problems	3.9 (2.0)	3.8 (2.0)	−0.21	.83
Hyperactivity	6.3 (2.3)	5.6 (2.4)	−1.88	.06
Peer problems	2.4 (1.4)	2.4 (1.6)	−0.01	.99
Prosocial behaviours (positive scale)	6.8 (1.8)	7.1 (1.8)	0.78	.44

## Discussion

This study entailed a comprehensive examination of the psycholinguistic and socioemotional profiles of young people in the youth justice system. It is the first study conducted in the United Kingdom to report on the literacy and socioemotional profiles of females in the Youth Justice System, and it generated the largest sample to date to have examined the point prevalence of unidentified DLD in young people on community, or triage, orders.

### Prevalence of DLD, gender, and profiles of female offenders

Consistent with previous research, the young people in this study demonstrated poor language skills. Operationalizing DLD as 1.5 *SD*s below the population mean on the expressive and/or the receptive CELF‐4 subtest meant that 87 participants (60% of the sample) met criteria for DLD diagnosis. For these participants, their DLD was unidentified with only two participants stating they had previously seen a speech and language therapist. These two participants, however, did not know why they had previously been a recipient of speech and language therapy services, although one suggested he had difficulty speaking when he was much younger. No participant was currently accessing speech and language therapy services, and of the 36 participants who had an education, health, and care plan, none detailed speech, language, and communication needs as a primary need (behaviour, emotional and social difficulties, attention‐deficit hyperactivity disorder, and autism were the non‐mutually exclusive categories recorded).

Approximately 20% of young offenders in England and Wales are female (YJB, 2018), so the proportion in this sample (23%) was broadly representative of the national picture. As mentioned above, 87 young offenders met criteria for DLD diagnosis. DLD was as prevalent in female youth offenders (*n* = 22, 67%) as it was in males (*n* = 65, 58%). Although international research has considered female offending populations, our UK point prevalence is considerably higher than that reported by researchers undertaking research in other countries. For example, Snow *et al*. (2016), in Australia, detailed a prevalence rate of 27% while Sanger, Moore‐brown, Magnuson, and Svoboda in the United States (2001) reported a prevalence rate of 19%. Even though methodological and cultural variations are likely to be responsible for some of the variation in prevalence rates, they all report higher proportions than what would be expected in the general population. Previous research has highlighted the need for consideration of such high levels of DLD when interventions in the YJS are being planned and delivered (Hughes *et al*., 2017; Snow *et al*., 2016). Our findings concur with this approach and extend this to the population of young female offenders.

It is also noteworthy that the majority of participants with DLD, irrespective of their gender, revealed severe language difficulties. Furthermore, among the 87 participants identified as having DLD, only two reported previously accessing speech and language services when in primary education. This means that the needs of 85 young people had previously gone unrecognized and they had not accessed any support. As Bryan *et al*. (2015) pointed out in their compounding risk model, this lack of identification of language needs is of concern particularly when considering there are potential opportunities to intervene earlier with young people who are struggling or disengaged from education. Bryan and colleagues’ model draws upon the associations of DLD with poorer outcomes in multiple domains including, but not restricted to, literacy, educational attainment (Conti‐Ramsden & Durkin, 2012), employment (Johnson, Beitchman, & Brownlie, 2010), anxiety disorders (Wadman, Botting, Durkin, & Conti‐Ramsden, 2011), and problematic behaviours (Yew & O'Kearney, 2013). The authors suggest that these multiple risks allow for points of intervention and advocate that young people with complex profiles, such as behavioural difficulties and disengagement from education, should be prime targets for language assessment and intervention (Bryan *et al*., 2015). Underlying shared risk factors may be responsible for these associations or they could be secondary difficulties due to the impact of DLD. Proficiency with language is essential for interacting with the world from initiating and maintaining friendships (Durkin & Conti‐Ramsden, 2007) to engagement with learning (Conti‐Ramsden, Botting, Simkin, & Knox, 2001). Most of this research concentrates on individuals with a history of identified DLD. Unfortunately, it is not known whether undiagnosed DLD negatively shapes adolescents’ school and life experiences, and thus elevates the risk of young people becoming involved in criminality. Longitudinal evidence is needed to develop our knowledge and understanding in this area.

There were no significant gender differences in the psycholinguistic and socioemotional profiles of male versus female young offenders bar higher levels of emotional difficulties in females. This is in line with epidemiological studies that have reported females as being more likely to experience internalizing problems such as emotional difficulties (Rescorla *et al*., 2007). However, we did not find that male young offenders were more likely to report externalizing difficulties. Although this investigation included the largest sample of female young offenders in the United Kingdom, one must still consider the possibility that the number of females in the sample may have limited the study's power to identify gender differences in small effect size. The clinical relevance of potential gender differences in small effect size needs to be carefully considered prior to any future research in this area.

### Associated reading difficulties

The mean chronological age scores gained in the sight word reading and phonemic decoding were, respectively, almost 4 and 3 years, on average, behind that of the participants’ actual ages. Qualitative observations during testing revealed that many young people began the session by declaring that they did not want to do any reading, with the majority remarking they found reading very difficult. Indeed, the proportion of participants who either refused or were unable to complete the reading tasks was high. The strenuous nature of engaging in reading activities could also affect motivation to engage with print. When measuring print exposure and reading skill, Harlaar and colleagues reported that independent reading at age 10 did not significantly predict reading achievement at 11. This was not the case for the cross‐lagged relation as reading achievement at age 10 predicted independent reading at age 11. This suggests the effect ran from reading skill to print exposure between the ages of 10 and 11 years (Harlaar, Deater‐Deckard, Thompson, DeThorne, & Petrill, 2011). Likewise, a recent study utilizing direction of causality models concluded that it was reading ability driving print exposure (Van Bergen *et al*., 2018).

Reading single words and phonemic decoding was an area of difficulty for the group, and qualitative observations confirm reading appeared effortful and laborious. It is possible that the lack of automaticity when reading single words leads to a superfluous amount of cognitive resources used leaving fewer resources for comprehending the text. In this study, 26 young people refused the reading comprehension task and a further 19 abandoned the task following the inability to correctly answer any of the first five questions. A further 42 participants did not begin the task due to a lack of time or a failure to attend a second appointment. As all these participants were excluded from the analysis, the results gained are likely to be an overestimation of the reading abilities of young offenders and caution should be exercised before generalizing the reported findings. The data reported here strongly suggest that the young offenders are likely to find reading youth justice‐related documentation difficult. This could potentially impact a young person's ability to engage with the YJS in terms of attending appointments, adhering to orders and service behaviour agreements. They also indicate that the individuals with DLD have significantly worse reading skills than those without DLD and, are therefore, likely to be further disadvantaged. There is a place for further research in this area. The poorer results obtained in the DLD group for both literacy and NVIQ could suggest difficulties in areas such as working memory and executive functioning.

### Psycholinguistic and socioemotional profiles of young offenders

Alexithymia was not distinctively associated with DLD. These data reinforce evidence from Snow *et al*. (2016) who suggest that alexithymia and DLD, in forensic populations, appear to be co‐morbid conditions as opposed to a complex single language factor tapping a variety of skills. Nonetheless, results of this investigation also revealed that alexithymia was a notable difficulty or co‐morbid condition of young offenders. Alexithymia was over‐represented in the sample as a whole with nearly two thirds (60%) of the participants who completed the TAS‐20 meeting criteria for having ‘alexithymia’ or ‘possible alexithymia’. The socioemotional profiles of participants were assessed via self‐report questionnaires. Although test items were read aloud to participants and, if required, were repeated, it is acknowledged that this places pressure on auditory processing skills which could weaken the validity of the tests.

The prevalence in the general population is 10% (Salminen, Saarijarvi, Toikka, & Karhanen, 1999), although it has been suggested it is higher in the adolescent population with approximately 24% of ‘normal adolescents’ scoring in the alexithymic range (Horton, Gewirtz, & Kreutter, 1992). Alexithymia is often described as a deficit in recognizing, experiencing, and processing emotions (Taylor, 1997), including an externally orientated thinking which has been referred to as a tendency to avoid affective thinking and viewing events superficially (Franz *et al*., 2008). These difficulties have the possibility to confer vulnerability for poor social exchanges and lack of prosocial behaviours in young people.

Our findings indicate that youth justice staff should be aware of the difficulties young offenders face in both their language processing skills and their ability to identify and label emotional states in themselves and others. Poor skills in these areas are likely to leave young people compromised in their ability to engage in rehabilitative strategies that are key to RJ processes. Reading difficulties and poor literacy skill more generally are likely to be implicated. The findings with regard to reading reported in this study reveal that those young offenders with DLD are more strongly disadvantaged than those without. Data from semi‐structured interviews with victim liaison officers support this notion. Results revealed that, even if a young offender writes a letter of apology, it is not always made available to the victim (Newbury, 2011). Staff highlighted that despite the young person putting in considerable effort in letter writing, the brevity of the letter could be perceived by victims as an insult (Newbury, 2011). This is analogous to the monosyllabic responses a young person with DLD may offer with face‐to‐face RJ. If accompanied by non‐verbal behaviours such as poor eye contact, it is possible victims could perceive these behaviours as rudeness or indifference (Snow & Powell, 2011). In the same vein, an evaluation of youth justice triage services identified shortcomings in the ability of the young people to complete workbooks or write letters of apology to victims (Soppitt & Irving, 2014). The time constraints and number of measures covered precluded a comprehensive writing assessment, and this is an area that warrants future research.

### Concluding remarks

Previous research has mainly focused on incarcerated (Bryan *et al*., 2007; Hughes *et al*., 2017) young offenders or those on intensive community orders (Gregory & Bryan, 2011). This study provides novel evidence pertaining to the needs of young people new to the YJS and allows for the application of this knowledge when planning intervention and rehabilitative programmes. Findings revealed a high degree of need among first‐time entrants into the YJS and furthermore specified key deficits in this population. Both DLD and alexithymia were found to be over‐represented in young offenders and equally prevalent in females and males. In addition, over half of young offenders exhibited socioemotional difficulties in the abnormal/borderline range and once again these difficulties were generally not gender‐specific. It is important to note that these data are in many ways ‘the best possible scenario’ for the participants due to the following reasons. Although the CELF‐4 is a standardized instrument that provides vital information regarding linguistic skills, performance on such a task in a quiet room may not reveal as many problems as those young people may experience in real life, when competing demands are included. In the context of RJ, demands will be placed on working memory as young people need to sustain attention and process the language of others in real time (Snow, Powell, & Sanger, 2012). Additionally, verbally mediated executive functions including planning and response inhibition will be utilized (Snow & Sanger, 2011). One would expect this to occur during high levels of stress (Snow & Sanger, 2011). In a similar vein, the language measures utilized focus on the structural aspects of language as opposed to targeting high‐level language skills such as non‐literal language comprehension and inferencing skills (Adams, 2002). Future research should consider such sociocognitive and pragmatic skills, including awareness of listener prior knowledge and the ability to reflect and self‐correct. Overall, our findings point to the need for language assessment and identification of DLD as a crucial part of criminal justice services and potential priority for intervention in first‐time young offenders.

## Supporting information


**Appendix S1.** Supplementary materials.
