# Supplementary material for: Psycholinguistic and socioemotional characteristics of young offenders: Do language abilities and gender matter?
Source: Legal Criminol Psychol. 2019 Apr 5;24(2):195–214. doi: 10.1111/lcrp.12150 (PMC6767454; doi:10.1111/lcrp.12150)
Supplement: Supplementary file 1 — Appendix S1. Supplementary materials. [file LCRP-24-195-s001.docx]

Supporting information (for online publication only)

Further information on participants

G power software was used to estimate the appropriate number of participants required for a statistically significant effect. Incorporating an independent means design with an alpha level of 0.05 and based upon medium effect sizes it is estimated that a group of 51 would be needed to detect power. Therefore, some of the groups are lacking in power (male versus female), whereas other groups are sufficiently large to detect a moderate effect (DLD versus non-DLD).

Further information on participant recruitment

Following discussions with managers in local community youth offending services in the North West of England, 4 YOTs and a triage centre agreed to be involved in the research. In total 181 young people were recruited via their YOT caseworker, although only 102 attended their appointment. The reasons for non-attendance included: the young person deciding not to participate, the YOT caseworker did not inform the young person of the appointment, or the young person was missing from home or in police custody at the appointment time. Justifying the need for obtaining personal, sensitive data for young people who failed to attend was difficult, therefore we do not have information for these young people. As triage interventions consist of a single contact with young people, participants were recruited by way of a letter to the home address, sent via the triage centre. Each young person was made aware that a researcher would be at the triage session on the day and participants were asked to complete the consent form if they agreed to participate. In total the researcher attended 70 sessions over a 10-month period and 43 participants agreed to participate from this route. It is important to note that this investigation was based on voluntary participation, potentially limiting generalisability. On the one hand, in the sampled YOTs only half of the young people referred by caseworkers attended their appointments, and so only the young people who were confident that they could undertake the assessments may have attended. On the other hand, the converse could be true. As caseworkers were responsible for referring young people, it is possible they could have only referred the young people whom they had concerns about as opposed to all eligible individuals on their caseload.

The final sample included in this investigation comprised 145 young people, 96 of whom were first time entrants into the youth justice service in the North West of England. The mean age of the participants was 15.8 (*SD* 1.5) and their ages ranged from 12 to 17 years. Most of the 145 participants (*n*=112) were male. This reflects the latest national results that were published in 2017, which reported that 80% of first time entrants into the YJS were male (80%) and their mean age was 15.2 (Youth Justice Board, 2018). These figures, however, do not include young people who are subject to triage orders.

**Further information on procedures**

This study was conducted as a cross-sectional design. The lead researcher (MW) tested all participants in a private session at which parents and youth offending team staff were encouraged to attend. Despite the intention to hold two separate sessions for participants to complete the measures, this was not always possible for two reasons. First, young people often failed to attend a subsequent appointment and, second, only one contact was possible with the participants recruited via the triage centre. For the participants who did provide complete data in a single session, due care was taken that it did not last longer than 60 minutes and that frequent breaks were taken. Participants were either seen at the youth justice setting, local health centres, their care home or their school. Prior to each session adolescents were given explanations regarding the study and the tasks were presented in pictorial format on a laminated card. This explanation emphasised the confidentiality of all data collected, and the option of voluntarily withdrawing from the research at any moment. Young people were advised that they could abandon a task at any time or skip any tasks that made them feel uncomfortable. Moreover, participants were encouraged to disclose their own opinion and were reassured that there were no right or wrong answers. To aid compliance some participants determined task order by choosing the corresponding picture of the task, and once completed, the picture was removed. Others gave no preference, and for these young people the tasks were administered in a random order. Participants were often reminded that they could remove pictures at any time, including before or during a task. This happened regularly and not all participants were willing to complete all tasks. For some participants the self-rating scales were administered in an interview format due to literacy problems. Following assessment, a report was compiled for the young person’s caseworker detailing results and offering recommendations regarding adapting the intervention. If deemed necessary onward referral was advised and each YOT and the triage organisation had a referral pathway to local services that could not be provided in-house.

Further information on measures and statistical analyses

The following battery of tests allowed for a detailed assessment of the young person’s abilities, in multiple domains, with reference to the expectations of a forensic setting. Due to the hard to reach nature of the study population certain considerations had to be prioritised, when selecting the assessment battery. To be accepted by gatekeepers, the assessments needed to be expeditious with good face validity. Assessment burden was a main consideration as previous studies, with multiple measures, have reported high refusal rates (Bryan et al, 2015).

Standardised psycholinguistic measures

Nonverbal IQ

To assess nonverbal IQ (NVIQ) we administered the performance subscale of The Wechsler Abbreviated Scale of Intelligence (WASI, Wechsler, 1999). This test has norms for individuals aged 6 to 89 years. The reliability of the Performance IQ scale for the age range 12 – 16 range from .84 to .93 for block design and .86 to .96 for matrix reasoning. The reliability coefficients for the age 17 are slightly higher. Validity studies of the WASI reported in the manual provide evidence that the test is a valid quick screening measure of intellectual functioning.

Language

Two subtests of the Clinical Evaluation of Language Fundamentals (CELF-4^uk)^ (Semel *et al.* 2006), a standardised assessment, normed up to age 21 years 11 months, were utilised to assess language skills. The first subtest, ‘formulated sentences’ (FS), requires the young person to formulate a sentence, including a given word, based on a picture shown. The CELF-4 manual details the reliability of FS to be .82. Second, as a receptive measure, the subtest understanding spoken paragraphs (USP) was chosen. This subtest, with a reliability of 0.75, provides a standardised score pertaining to the young person’s ability to process, comprehend and formulate a response to factual and inferential information that has been delivered verbally. This mirrors what is expected of a young person in a forensic setting and was therefore seen to be a good window to the young person’s ability. Due to the expectation that the sample would consist mainly of young people from areas of low SES, we did not utilise a vocabulary assessment from the CELF-4. Vocabulary assessment is open to bias due to the reliance on experiences and exposure and understanding of school curricula (Spencer et al, 2012).

In an effort to avoid over-diagnosis, and following recommendations made in the review by Spencer, Clegg & Stackhouse (2012), a score of 1.5 *SD* below the normative mean on the CELF-4 subscales was used to determine the frequency of unidentified DLD. This resulted in 87 participants (60%) meeting the criteria of a scaled score of 77 or less on the expressive and/or receptive subtest. No significant gender difference in the prevalence of DLD, χ^2^ (1) = 7.91, *p* = 0.37 was found. Of the 112 males in the study, 65 (58%) met the criteria as did 22 (67%) of the females. The young people showed a variety of different language profiles with the majority, 55 participants (38%), gaining scores indicative of having both expressive and receptive DLD. A smaller proportion, 12 participants (8%) displayed receptive difficulties only and 20 participants (14%) returned scores indicative of difficulties with the expressive domain. The Table below demonstrates that the largest proportion of those with language difficulties returned scores on the CELF-4 subtests in the ‘severe’ range.

**Table.** Group results by severity of expressive and receptive language

| CELF-4 subtest severity score | Expressive measure (FS) | Receptive Measure  (USP) |
| --- | --- | --- |
| Average (86-114) | 59 (41%) | 63 (44%) |
| Mild (78-85) | 11 (8%) | 15 (10%) |
| Moderate (71-77) | 17 (11%) | 5 (3%) |
| Severe (70 and below) | 58 (40%) | 62 (43%) |
| Total | 145 (100%) | 145 (100%) |

Reading

The Test of Word Reading Efficiency–Second Edition (TOWRE–2; Torgeson, Wagner & Rashotte, 1999) was utilised as a measure of an individual’s ability to pronounce printed words (Sight Word Efficiency) and non-words (Phonemic Decoding Efficiency) accurately and fluently. This test requires the young person to read words and non-words (readable letter combinations without any meaning, for example ‘barp’) from a card as fast and accurately as possible. Only the words read accurately were considered as correct and mispronunciations were counted as errors unless the young person self-corrected. The manual suggests when internal consistency was investigated the subtests and the total scores all exceeded the .80 level (Torgeson et al, 1999).

Reading comprehension was measured using the Wechsler Individual Achievement Test (WIAT-II; Wechsler, 2005). This includes a subtest with stories and sentences assessing literal, inferential and lexical comprehension. Strong inter-item consistency within subtests, are reported, with average reliability coefficients ranging from .80 to .98.

Socio-emotional measures

Alexithymia

To measure Alexithymia, we used The Toronto Alexithymia Scale (TAS-20; Bagby, Taylor & Parker, 1994). The TAS-20 is a self-report scale that is comprised of 20 items, measuring the following three subscales: 1) difficulty describing feelings (‘It is difficult for me to find the right words for my feelings’); 2) difficulty identifying feelings (‘When I am upset I don’t know if I’m sad, frightened or angry’); 3) externally-orientated thinking (‘I prefer to just let things happen, rather than understand why they turned out that way’). Items are rated using a 5-point Likert scale whereby 1 = ‘strongly disagree’, 2 = ‘disagree’, 3 = ‘neutral’, 4 = ‘agree’ and 5 = ‘strongly agree’. There are 5 items that are negatively worded. Although alexithymia can be conceptualised as a dimensional construct an indicative cutoff score has been established empirically, facilitating comparisons between studies (Taylor, 1997). We used this total alexithymia score, which is the sum of responses to all 20 items, and scoring is described as; equal to or less than 51 = non-alexithymia, 52 to 60 = possible alexithymia and scores equal to or greater than 61 = alexithymia.

External and internalising difficulties

To measure the presence of external and internalising difficulties we used the Strengths & Difficulties questionnaire (SDQ; Goodman, 1997). The SDQ consists of 25 statements covering 5 domains of functioning: conduct problems (e.g. ‘I get very angry’), hyperactivity (e.g. ‘I am easily distracted’), emotional difficulties (e.g. ‘I worry a lot’) peer relation problems (e.g. ‘I am usually on my own’), and, prosocial behaviour (e.g. ‘I try to be nice to others’). The latter scale, the prosocial scale, measures positive functioning (as opposed to difficulties). For each item, the young person could tick either ‘not true’, ‘somewhat true’ or ‘certainly true’, which reflect a score of 0, 1 and 2, respectively. Some items are, however, reverse-scored such as, in the conduct problems scale, ‘I usually do as I’m told’. In the hyperactivity scale, ‘I think before I do things’ would also be reversed-scored with ‘not true’ scoring 2, and ‘certainly true’ scoring 0 (with ‘somewhat true’ always scoring 1).

Contextual measures

Offence characteristics

Detailed scrutiny of departmental files in each YOT and the triage centre took place to ascertain the nature of the offence the young person had committed.

Offender characteristics

Offender characteristics were extracted from the file, including education provision, looked after status, whether the young person was in education, employment or training, had any recorded special need or an Education Health and Care plan, a history of missing from home, truanting or self-harm.

Educational attainment: Literacy and numeracy

On arrival to the YOT, staff routinely liaised with a young person’s educational establishment to document current level of attainment. Standard Assessment Tests (SATs) are mandatory assessments in the UK, that occur at intervals across the school years, and are designed to measure progress against the national curriculum. The information was recorded following the levels from the Qualifications and Credit Framework (QCF), which operates a system of grouping qualifications according to difficulty. The framework ranges from entry level, which encompasses three ranks and culminates at level 8 - the highest level. Entry level qualifications (1-3) refer to basic knowledge and skills and are designed for students who are not ready for GCSEs. The key milestones for the age of this sample are entry level 3, which children are expected to achieve at the end of Key Stage 3 (KS3) and level 1, which denotes a GCSE grade D-G and level 2 which denotes a GCSE grade A*-C. The highest level of academic qualification was obtained by YOT staff and recorded in the young person’s file. Data concerning the literacy and numeracy educational attainments of the participants were extracted from the participating organisations’ databases.

Statistical Analysis

To avoid losing statistical power, missing values on the questionnaires were dealt with using a pro-rating method. In this method each missing value was replaced with the mean of the non-missing values for the same question from the same participant’s data (Field, 2005). The maximum number of missing responses for any participant was two out of a possible 20 responses. Responses were missing because the participant had failed to answer the question, or the participant’s response was unintelligible. A speech and language therapist, independent of the study, scored a random sample of 20% of the formulated sentences sub-test. The inter-rater reliability analysis revealed adequate reliability Kappa = 0.83, *p* = < 0.001. Inter-rater reliability was not deemed necessary for the understanding spoken paragraphs sub-test as the marking booklet contains prescriptive answers.
